# Incidence of Homozygous *SMN2* Deletion in Japan: Cross-Reactivity of *SMN2* Primers with *SMN1* Sequence Causes False Negatives in Real-Time PCR Screening

**DOI:** 10.3390/genes16060712

**Published:** 2025-06-16

**Authors:** Makoto Sakima, Yoshihiro Bouike, Shin-Ichi Wada, Masami Nakamae, Yoriko Noguchi, Ryosuke Bo, Hiroyuki Awano, Jumpei Oba, Hisahide Nishio

**Affiliations:** 1 Faculty of Nutrition, Kobe Gakuin University, 518 Arise, Ikawadani-cho, Nishi-ku, Kobe 651-2180, Japan; n8yemx03@s.kobegakuin.ac.jp (M.S.); bouike@nutr.kobegakuin.ac.jp (Y.B.); shin1wada@nutr.kobegakuin.ac.jp (S.-I.W.); mnakamae@nutr.kobegakuin.ac.jp (M.N.); 2 Department of Clinical Laboratory, Kobe University Hospital, 7-5-1 Kusunoki-cho, Chuo-ku, Kobe 650-0017, Japan; ynoguchi@med.kobe-u.ac.jp; 3 Department of Pediatrics, Graduate School of Medicine, Kobe University, 7-5-1 Kusunoki-cho, Chuo-ku, Kobe 650-0017, Japan; ryobo@med.kobe-u.ac.jp; 4 Organization for Research Initiative and Promotion, Research Initiative Center, Tottori University, 86 Nishi-cho, Yonago 683-8503, Japan; awano@tottori-u.ac.jp; 5 Department of Occupational Therapy, Faculty of Rehabilitation, Kobe Gakuin University, 518 Arise, Ikawadani-cho, Nishi-ku, Kobe 651-2180, Japan; j-oba@reha.kobegakuin.ac.jp

**Keywords:** spinal muscular atrophy, *SMN1*, *SMN2*, real-time PCR, PCR-RFLP, nucleotide sequencing analysis, false positive, false negative, cross-reactivity

## Abstract

**Background**: *SMN1* and *SMN2* are causative and modifier genes, respectively, for spinal muscular atrophy (SMA). The incidence of *SMN1* homozygous deletion in Japan is 1 in 20,000. However, the incidence of *SMN2* homozygous deletion in Japan remains unknown. **Methods**: To clarify the incidence of homozygous *SMN2* deletion in Japan, real-time polymerase chain reaction (PCR) was performed on dried blood spot (DBS) samples collected from newborns nationwide. Samples with positive or ambiguous results were retested using PCR-restriction fragment length polymorphism (PCR-RFLP) and nucleotide sequence analysis. **Results**: Of the 1000 DBS samples that were screened using real-time PCR, 51 were positive. Retesting using PCR-RFLP analysis identified 10 false results: six false positives and four false negatives. Therefore, there were 49 true positives among the 1000 samples. Notably, nucleotide sequence analysis revealed that the false negatives were caused by the cross-reactivity of *SMN2* primers with *SMN1* sequences. **Conclusions**: The incidence of homozygous *SMN2* deletion in Japan is approximately 1 in 20 people. This incidence is much higher than that of homozygous *SMN1* deletion and may reflect the vulnerability of the *SMN2* region. Importantly, the results of the present study suggest that false negatives in the screening process were caused by cross-reactivity with non-target gene sequences.

## 1. Introduction

Spinal muscular atrophy (SMA) is a lower motor neuron disease (LMND) that is inherited in an autosomal recessive manner [1]. In 1995, Lefebvre et al. [2] identified *SMN1* and *SMN2* as SMA-related genes on chromosome 5q13. *SMN1* and *SMN2* are nearly identical genes that are located in the SMA locus. However, only *SMN1* is a causative gene for SMA. More than 95% of SMA patients completely lack *SMN1* (i.e., homozygous *SMN1* deletion); the remaining patients may have point mutations or short deletions [2]. In contrast, more than 4% of control individuals lack *SMN2* (i.e., homozygous *SMN2* deletion) [2]. On the basis of genetic analysis, the incidence of SMA has been reported as 1 in 10,000–20,000 newborns, and the carrier frequency is 1 in 50–70 [3]. In Japan, the disease incidence is 1 in 20,000 [4,5].

The protein product of *SMN1* is a full-length survival motor neuron protein (SMN), which has been known to exert a wide variety of cellular functions [6]. On the other hand, the protein products of *SMN2* are the full-length SMN protein and an SMN protein lacking the exon 7 domain (Δ7-SMN). Full-length SMN is a minor product of *SMN2*, whereas Δ7-SMN is a major product; however, Δ7-SMN is unstable and non-functional [6].

Why is *SMN2* considered a modifier gene for SMA? No homozygous deletion of *SMN2* has been identified in SMA patients, supporting the hypothesis that the complete loss of *SMN1* and *SMN2* would result in embryonic or fetal lethality [6]. Thus, even small amounts of full-length SMN produced by *SMN2* might be essential for embryonic or fetal survival in SMA patients. More importantly, SMA patients who have higher copy numbers of *SMN2* exhibit ameliorated phenotypes [7]. *SMN2* may therefore have a role in compensating for SMN1 loss (or deleterious mutation), at least to some degree.

The other roles of *SMN2* in neurological diseases are largely unknown. Moulard et al. [8] identified homozygous *SMN2* deletion in 36% of individuals with sporadic adult-onset LMND. Furthermore, Srivastava et al. [9] and Liping et al. [10] suggested that homozygous *SMN2* deletion may cause distal muscle disorders. However, the relationship between homozygous *SMN2* deletion and neurological disease remains a subject of long-standing debate.

Anthropologically, the frequency of *SMN2* deletion is of great interest. Vorster et al. [11] reported a surprisingly high frequency of homozygous *SMN2* deletion in South Africa. They identified homozygous *SMN2* deletion in 12% of Black controls and 30% of Black patients with SMA-like symptoms. The authors suggested that these data may reflect architectural changes in the SMN region, including novel gene conversion or rearrangement events.

To clarify the relationship between homozygous *SMN2* deletion and neurological diseases or the architectural stability of *SMN2* in the SMA locus, it is essential to determine the incidence of homozygous *SMN2* deletion in each ethnic group. Given that no epidemiological studies on homozygous *SMN2* deletion were conducted in Japan until 2003, we planned to establish a screening system for homozygous *SMN2* deletions. In 2023, we developed a real-time polymerase chain reaction (PCR) screening method to detect homozygous *SMN2* deletions [12].

As a preliminary step to clarify the relationship between homozygous SMN2 deletion and neurological diseases in Japan, it is necessary to determine the frequency (or incidence) of homozygous SMN2 deletion in the Japanese population. In the present study, we used our real-time PCR method to screen 1000 dried blood spot (DBS) samples collected from newborns nationwide to estimate the incidence of homozygous *SMN2* deletion in Japan. During the screening process, we encountered both false-positive and false-negative samples, which we also report in this article.

## 2. Materials and Methods

### 2.1. Residual DBS Samples

In the present study, 1000 residual DBS samples on filter paper (FTA Elute Cards; GE Healthcare, Boston, MA, USA) from newborn infants born all over Japan were used. The samples were randomly selected from residual samples that had been collected from 4157 newborn infants in our previous pilot study [13].

### 2.2. Real-Time PCR

**Figure 1:**
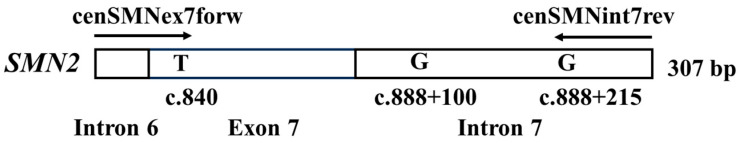
Primer positions used in the real-time polymerase chain reaction analysis. The forward primer (cenSMNex7forw) binds to the intron 6/exon 7 boundary, including the *SMN2*-specific nucleotide, c.840T. The reverse primer (cenSMNint7rev) binds to the intron 7 sequence, including the *SMN2*-specific nucleotide, c.888+215G. The third *SMN2*-specific nucleotide, G at c.888+100, exists in intron 7. When false-positive results caused by cross-reactivity with the *SMN1* sequence were obtained, the amplified products included the *SMN1*-specific nucleotide, A at c.888+100. This figure is reproduced from our previous report [12].

A punched circle (1.2 mm in diameter) from the DBS card was placed directly into a PCR mixture [12]. The components of the real-time PCR mixture and their concentrations are listed in Table S1. The primer sequences were cenSMNex7forw (5′-TTT ATT TTC CTT ACA GGG TTT TA-3′) [7] and cenSMNint7rev (5′-GTG AAA GTA TGT TTC TTC CAC GCA-3′) [7]. The primer positions are shown in Figure 1.

The PCR conditions were as follows: (1) initial denaturation at 94 °C for 7 min; (2) 30 cycles of denaturation at 94 °C for 1 min, annealing at 56 °C for 1 min, and extension at 72 °C for 1 min; and (3) a final extension at 72 °C for 7 min.

### 2.3. PCR-Restriction Fragment Length Polymorphism (RFLP)

**Figure 2:**
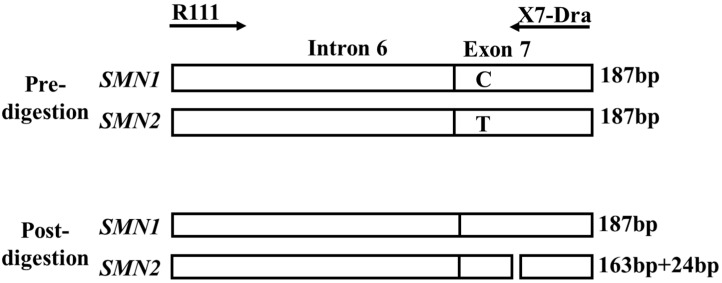
Primer positions used in the polymerase chain reaction (PCR)-restriction fragment length polymorphism analysis. The forward primer (R111) bound to intron 6 of *SMN1* and *SMN2*. The reverse mismatched primer (X7-Dra) bound to a region near c.840. The nucleotide at c.840 was G in *SMN1* and T in *SMN2*. A restriction enzyme, DraI, cut the PCR-amplified fragment of *SMN2* at c.840, creating two fragments (163 and 24 bp). This figure is a slightly modified version of a figure from our previous report [12].

To distinguish *SMN2* from *SMN1*, PCR-RFLP was performed according to the method reported by van der Steege et al. [14], with some modifications. The DNA sources in this study were DBS samples. The components of the PCR mixture and their concentrations are listed in Table S2. The primer sequences were R111 (5′-AGA CTA TCA ACT TAA TTT CTG ATCA-3′) [2] and X7-Dra (5′-CCT TCC TTC TTT TTG ATT TTG TTT-3′) [14]. The primer positions and restriction enzyme site are shown in Figure 2.

The PCR conditions were as follows: (1) an initial denaturation at 94 °C for 7 min; (2) 30 cycles of denaturation at 94 °C for 1 min, annealing at 56 °C for 1 min, and extension at 72 °C for 1 min; and (3) a final extension at 72 °C for 7 min. Thereafter, the amplified products were digested by restriction enzyme DraI at 37 °C for 12 h.

### 2.4. Nucleotide Sequencing

The real-time PCR products that had been amplified for 45 cycles in the screening procedures were purified using ISOSPIN PCR Product (NIPPON GENE Co., Ltd., Tokyo, Japan). The purified products were then submitted for sequencing. All sequencing analyses were performed by FASMAC Co., Ltd. (Atsugi, Japan).

The sequencing reactions were performed using an Applied Biosystems Big Dye Terminator V3.1 cycle-sequencing kit (Thermo Fisher Scientific Inc., Waltham, MA, USA). Reaction products were analyzed using an Applied Biosystems 3730xl DNA Analyzer (Thermo Fisher Scientific Inc.).

In the current study, we selected a direct sequence for sequencing DNA bases using products amplified directly by PCR. This eliminated the need for steps such as DNA cloning and plasmid purification and allowed us to directly read specific regions of the genome, which is useful when searching for genetic mutations.

### 2.5. Statistical Analyses

We used Excel software (version number 2025) (Microsoft Corp., Redmond, WA, USA) for the summary statistics. The cycle threshold (Ct) values of real-time PCR are presented as the mean ± standard deviation (SD). The mean Ct values of the *SMN2* deletion and *SMN2* retention groups were compared using Welch’s *t*-test. A *p*-value < 0.05 was considered significant.

## 3. Results

### 3.1. Detection of Homozygous SMN2 Deletion Using Real-Time PCR

We subjected 1000 DBS samples to real-time PCR, which is a suitable screening method because it can analyze many samples at once. The Ct value (mean ± SD) of 1000 samples was 32.7 ± 2.1.

**Figure 3:**
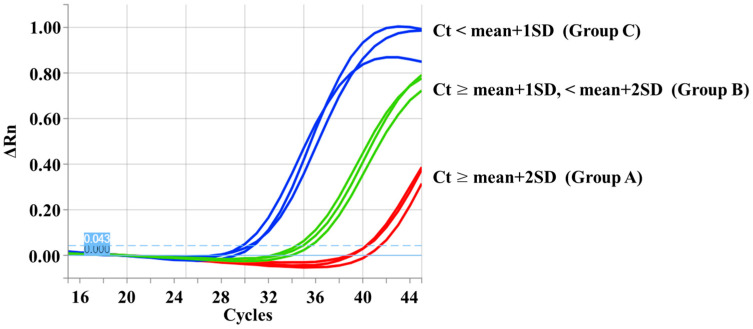
Real-time polymerase chain reaction amplification curves. The curves of representative cases from each group are shown.

The real-time PCR results were classified into three groups based on the Ct values (Figure 3): Group A, DBS samples with Ct values ≥ mean + 2 SD; Group B, DBS samples with Ct values ≥ mean + 1 SD and <mean + 2 SD; Group C, DBS samples with Ct values < mean + 1 SD.

Group A samples (*n* = 51) were most likely to be positive cases for homozygous *SMN2* deletion, Group B samples (*n* = 46) were ambiguous cases on the border between positive and negative results for homozygous *SMN2* deletion., and Group C samples (*n* = 903) were most likely to be negative cases for homozygous *SMN2* deletion.

### 3.2. Confirmation of the Real-Time PCR Results Using PCR-RFLP

Next, we retested the samples from Groups A and B (*n* = 51 + 46) using PCR-RFLP analysis. We did not retest the samples from Group C for two reasons: (1) there was a very small possibility that Group C included cases with homozygous *SMN2* deletion, and (2) given the time it takes to perform PCR-RFLP, we considered that there were too many samples in this group (*n* = 903) to feasibly retest using this method.

PCR-RFLP is relatively cumbersome for gene detection because it includes PCR amplification, enzymatic digestion, and agarose gel electrophoresis, meaning that it takes several hours or even half a day from start to finish. However, this method is very robust and produces stable results because sufficient PCR product can be obtained for subsequent RFLP analysis regardless of the original DNA quality and quantity from the DBS samples. Moreover, when the PCR products are completely digested, RFLP analysis provides accurate information about the presence or absence of the target gene.

**Figure 4:**
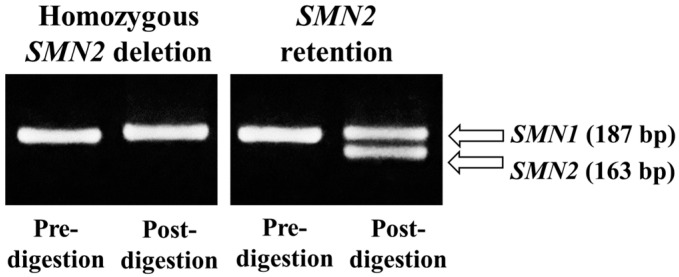
Polymerase chain reaction (PCR)-restriction fragment length polymorphism. The restriction enzyme DraI cuts the PCR-amplified fragment of *SMN2* exon 7 at c.840, creating two fragments (see Figure 2). The absence of 163-bp fragments indicates *SMN2* deletion.

To retest the samples with positive (Group A) and ambiguous (Group B) results, we conducted PCR-RFLP analysis using a second punched circle (near the first one) from each DBS. Figure 4 displays a representative gel electrophoresis result showing the digestion products with *SMN2* deletion and retention.

Table 1 summarizes the PCR-RFLP results in Groups A and B. Of the 51 DBS samples with Ct values ≥ mean + 2 SD (Group A), 45 had a homozygous *SMN2* deletion, whereas 6 retained *SMN2* alleles. Of the 46 samples with Ct values ≥ mean + 1 SD and <mean + 2 SD (Group B), 4 had a homozygous *SMN2* deletion, whereas 42 retained *SMN2* alleles. When Groups A and B were combined, 49 of the 97 samples had a homozygous *SMN2* deletion, and 48 samples retained at least one copy of *SMN2*.

### 3.3. Nucleotide Sequencing Analysis of the Real-Time PCR Amplified Products

After confirming the presence or absence of homozygous *SMN2* deletion using PCR-RFLP analysis, we conducted a nucleotide sequencing analysis using the real-time PCR-amplified products, which were obtained during the screening procedures.

All real-time PCR-amplified products could be subjected to nucleotide sequencing analysis. However, we only conducted a nucleotide sequencing analysis of the positive (Group A) and ambiguous (Group B) samples. We did not include the Group C samples in this analysis because of time and budget constraints. Even so, this nucleotide sequencing analysis clarified the cause of false-negative results and confirmed that the false-positive samples indeed retained *SMN2*.

**Figure 5:**
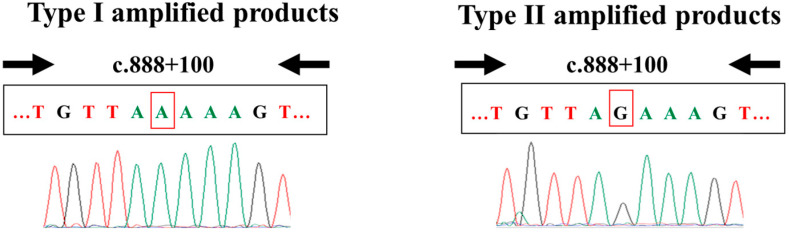
Type I and Type II amplified products. The primer set in this experiment generated two types of amplification products: Type I and Type II. (Left) Type I amplified products were amplified products that contained an *SMN1*-specific nucleotide: A at c.888+100. When true-positive or false-negative results (i.e., the absence of *SMN2*) were obtained, we only observed Type I products. (Right) Type II amplified products were amplified products that contained an *SMN2*-specific nucleotide: G at c.888+100. When true-negative or false-positive results (i.e., the presence of *SMN2*) were obtained, we only observed Type II products.

In this analysis, we identified two kinds of amplified products: Type I amplified products (with *SMN1* sequences) and Type II amplified products (with *SMN2* sequences) (Figure 5). The presence of Type I amplified products indicated that the *SMN2* primers had non-specifically amplified the *SMN1* sequence from samples with at least one copy of *SMN1*. The presence of Type II amplified products indicated that the *SMN2*-specific primers had specifically amplified the *SMN2* sequence from samples with at least one copy of *SMN2*. Of note, no mixed data (containing both types of amplified products) were obtained.

Table 2 summarizes the nucleotide sequencing results in Groups A and B. Of the 51 DBS samples with Ct values ≥ mean + 2 SD (Group A), 45 had a homozygous *SMN2* deletion (Type I), whereas 6 retained *SMN2* alleles (Type II). Of the 46 samples with Ct values ≥ mean + 1 SD and <mean + 2 SD (Group B), 4 had a homozygous *SMN2* deletion (Type I), whereas 42 retained *SMN2* alleles (Type II). When Groups A and B were combined, 49 of the 97 samples had a homozygous *SMN2* deletion, and 48 samples retained at least one copy of *SMN2*. These results were entirely consistent with the PCR-RFLP data (Table 1).

Collectively, our findings indicate that the false-negative results of the real-time PCR screening were caused by relatively high amplification caused by the cross-reactivity of the *SMN2* primers with the *SMN1* sequence. Additionally, the false-positive results of the real-time PCR screening were caused by relatively low amplification, which can be caused by low amounts of DNA in the sample and/or the presence of PCR-inhibiting compounds such as heparin. However, heparin was not used during sample preparation in the present study. The most plausible cause of false-positive results was therefore a low amount of DNA in the DBS samples.

### 3.4. Sensitivity and Specificity of the Screening, and Incidence of Homozygous SMN2 Deletion

In the present study, all DBS samples with Ct values < mean + 1 SD (Group C) were assumed to retain *SMN2*. Table 3 shows the sensitivity and specificity of real-time PCR in the 1000 samples in this study using the assumed data from Group C combined with the confirmed data from Groups A and B (Table 1). Although the 903 samples from Group C were not retested using PCR-RFLP, it is considered highly unlikely that this group contained cases of homozygous *SMN2* deletions. We thus determined a sensitivity of 0.918 (45/49), specificity of 0.994 (945/951), positive predictive value of 0.882 (45/51), and negative predictive value of 0.996 (945/949).

Using these data, we defined two new groups based on the absence/presence of SMN2: homozygous SMN2 deletion and SMN2 retention. The Ct values for the homozygous SMN2 deletion and SMN2 retention groups were 39.2 ± 1.9 (*n* = 49) and 32.4 ± 1.5 (*n* = 951), respectively. The Ct values were significantly different between the two groups (*p* < 0.01). However, in the actual screening situation, the Ct values for both groups had a small overlap caused by false results, as described in the previous sections.

Finally, using the real-time PCR results and the subsequent close examination of ambiguous cases using PCR-RFLP and nucleotide sequencing, we determined the incidence of individuals with homozygous *SMN2* deletion in Japan as 49/1000 (4.9%) (Table 4).

## 4. Discussion

### 4.1. Establishment of a Screening System for Homozygous SMN2 Deletion

Our previous study was performed to develop a methodology for detecting homozygous *SMN2* deletion using real-time PCR [12]. To develop a methodology, we first needed to identify positive samples (i.e., those with homozygous *SMN2* deletion). We therefore tested 300 newborn screening (NBS) samples using PCR-RFLP. However, the PCR-RFLP method is relatively time-consuming and laborious, and it is unsuitable for screening many samples. It was therefore necessary to establish a simple, rapid, and easy method using real-time PCR.

The present study was performed to screen more samples (1000 samples) using the real-time PCR method that was developed in our previous study. Because we faced problems (especially false results) in the screening process using real-time PCR, we retested some samples using PCR-RFLP and used nucleotide sequence analysis to identify the cause of false results (see Section 4.3). Finally, we estimated the incidence of homozygous *SMN2* deletion in Japan as approximately 5% in the general population (see Section 4.2).

In our series of studies, we chose the direct PCR method using intercalating fluorescent technology because of its speed, simplicity, and low cost. Our screening system included no DNA extraction step (speed and simplicity), no fluorescent probes (simplicity and low cost), or no reference gene analysis (simplicity and low cost).

To avoid punch-to-punch variability, DNA should have been extracted from DBS in the first step of the analysis. However, this is time-consuming and requires many punched DBS circles. The direct PCR method allows us to skip the step of DNA extraction from DBS, thus saving DBS samples and completing the screening assay quickly.

To co-amplify the reference gene, we should have used the fluorescent probe method (5′ nuclease assay), but fluorescent probes are expensive. The intercalating fluorescent technology in our studies was unable to detect two or more different genes specifically, but intercalating fluorescent dyes are inexpensive. In addition, if one only aims to screen for the presence or absence of a target gene, introducing a reference gene may not be necessary, because screen-positive cases (i.e., target-gene-absent cases) or ambiguous cases should be retested, which was demonstrated in this study.

### 4.2. Incidence of Homozygous SMN2 Deletion in Japan

We screened DBS samples from newborns in Japan for homozygous *SMN2* deletion using a real-time PCR method with an intercalating fluorescent dye. In our study, 951 of the 1000 DBS samples carried at least one copy of *SMN2*, whereas 49 samples had a homozygous *SMN2* deletion. Our findings, therefore, indicate that the incidence of homozygous *SMN2* deletion in Japan is 49 in 1000, or 4.9% (Table 4). The incidence of homozygous *SMN2* deletion in Japan is similar to that in other Asian countries, slightly lower than that in European countries, and much lower than that in African countries (see Section 4.5).

### 4.3. False Results Identified in the Present Study

In the current study, Ct values ≥ mean + 2 SD were considered positive for homozygous *SMN2* deletion, whereas Ct values < mean + 2 SD were considered negative for homozygous *SMN2* deletion. To detect false-negative and -positive results, we also performed PCR-RFLP analysis for all samples with Ct values > mean + 1 SD. PCR-RFLP analysis identified six false-positive samples and four false-negative samples out of 1000 DBS samples. Subsequent nucleotide sequencing analysis demonstrated that false-negative results were caused by cross-reactivity with the *SMN1* sequence.

Our real-time PCR method used specific primers to detect the target gene. However, although both the sense and antisense primers used in the present study were designed specifically for the target gene [7], false negatives still occurred. To prevent false negatives, future studies may consider the use of locked nucleic acid-modified primers [15]. In previously reported real-time PCR methods using specific probes, locked nucleic acid-modified probes have been used to increase the stringency of probe hybridization [16].

False positives are highly dependent on the condition of the DBS sample, including the coexistence of anticoagulants such as heparin [5,12]. To improve the accuracy of the screening system, attention should therefore be paid to sample preparation.

### 4.4. Possible Pitfalls of Neonatal SMN1 Deletion Screening

For the early detection of SMA, NBS programs to detect *SMN1* deletion (SMA-NBS) are being implemented around the world [17], and real-time PCR analyses are used in many SMA-NBS laboratories [18].

A systematic review by Cooper et al. [18] noted that most previous SMA-NBS studies reported no false-negative or -positive cases (i.e., they had a sensitivity and specificity of 100%). The authors suggested that some false negatives may have been missed because of a lack of follow-up for the negative results. When a screening test determines that *SMN1* is absent (i.e., the case is positive for SMA), retesting is usually performed to avoid false-positive cases. However, when a screening test determines that *SMN1* is present (i.e., the case is negative for SMA), retesting is usually skipped, meaning that false-negative samples containing *SMN1* may be overlooked, although this is very rare.

Importantly, the pitfalls of screening tests that were identified in the present study, particularly regarding false-negative results, may also exist in SMA-NBS.

### 4.5. Anthropological Importance of SMN2 Deletion

A large-scale gene duplication in primates gave rise to a variety of paralogous genes [19]. Human *SMN2* is one such paralogous gene; it was created by the duplication of an *SMN1*-including segment approximately three million years ago [19]. The difference between human *SMN1* and *SMN2*, which are both approximately 20 kb in size, was initially thought to be only 5 bp [2] but was later reported to be at least 22 bp [20]. Nonetheless, *SMN1* and SMN2 are almost identical genes. This high homology makes the SMA locus on the chromosome unstable, which leads to genomic instability and a predisposition to gene deletions, duplications, and conversions between both *SMN1* and *SMN2* [20].

Table 5 shows the frequencies of homozygous *SMN2* deletion around the world, demonstrating that more than 10% of Sub-Saharan individuals have a complete absence of *SMN2*. Notably, Sub-Saharan individuals are likely to have three or more copies of *SMN1*, and the carrier frequency of SMA is much lower in this region than in other areas [11,21]. Furthermore, a high copy number of *SMN1* in conjunction with a lack of *SMN2* is relatively common in Black South African individuals, whereas a low copy number of *SMN1* is relatively common in European/Asian individuals. These observations may be explained by the two following scenarios:

**Table 5:** Frequencies of homozygous *SMN2* deletion around the world *.

	Country/ Ethnicity	Controls		NeuromuscularDisorders		References
		*SMN2*-Deletion/ Sample Number	Frequency	*SMN2*-Deletion/ Sample Number	Frequency	
**European**						
1	UK	(-)	(-)	16/154	10.4%	[22]
2	USA and Canada	4/54	7.4%	(-)	(-)	[23]
3	France	8/90	8.9%	(-)	(-)	[24]
	France	15/167	9.0%	16/167	9.6%	[25]
4	France	52/621	8.4%	54/600	9.0%	[26]
5	Sweeden	37/502	7.4%	29/502	5.8%	[26]
6	Germany	9/100	9.0%	(-)	(-)	[27]
7	The Netherlands	78/984	7.9%	62/847	7.3%	[28]
**African**						
8	Sub-Saharan (Mali)	150/613	24.5%	(-)	(-)	[21]
9	Sub-Saharan (Nigeria)	33/120	27.5%	(-)	(-)	[21]
10	Sub-Saharan (Kenya)	23/120	19.2%	(-)	(-)	[21]
11	Black South African	15/122	12.3%	60/122	49.2%	[11]
**Asian**						
12	Vietnam	2/52	3.9%	(-)	(-)	[29]
13	Chinese	89/1712	5.29%	(-)	(-)	[30]
14	Taiwan	30/520	5.8%	(-)	(-)	[31]
15	Taiwan	5147/107,611	4.8%	(-)	(-)	[32]
16	Korea	2/100	2.0%	5/25	20.0%	[33]
17	Korea	49/1581	3.1%	(-)	(-)	[34]
18	Japan	16/300	5.3%	(-)	(-)	[12]
19	Japan	19/399	4.8%	41/537	7.6%	[35]
20	Japan	49/1000	4.9%	(-)	(-)	This study

* Homozygous deletion of *SMN2* exon 7, specifically; (-): no data reported.

(1) Vorster et al. [11] noted that primates have only one copy of *SMN1*, suggesting that the *SMN* region in early humans might have consisted of only the *SMN1* gene. At a later stage, the hypervariable nature of the *SMN* region might have resulted in multiple copies of *SMN1*. This duplicated *SMN1* might have then diverged into *SMN2* as a result of mutations. Thus, a high copy number of *SMN1* and a lack of *SMN2* may reflect a state before the divergence of *SMN2* from *SMN1*.
(2) Sangaré et al. [23] posited that the population that migrated out of Africa to Asia/Europe may have had a lower *SMN1* copy number by chance, or might have randomly drifted in this direction after the migration. Black South African individuals may therefore represent the descendants who retained genetic diversity, whereas European/Asian individuals represent the descendants with reduced genetic diversity caused by the bottleneck phenomenon. Thus, the bottleneck phenomenon might be the driving force underlying the differences in SMA-related genotypes between Black South African and European/Asian individuals.

### 4.6. Perspectives from SMN2 Studies

Studies using animal models indicate that the loss of both *SMN1* and *SMN2* may lead to fetal lethality [36,37], indicating that the presence of *SMN2* is critical for the survival of *SMN1*-deficient fetuses. This concept guided us to the idea that *SMN2* loss may be related to disease development in patients with some genetic defects.

In recent years, there has been debate regarding the relationship between homozygous *SMN2* deletion and motor neuron disease. Echaniz-Laguna and Lee [38] concluded that homozygous *SMN2* deletion is a risk factor for developing amyotrophic lateral sclerosis (ALS), whereas Corcia [26] suggested that it is a preventive factor against ALS progression in some ethnicities. However, Gamez [39] reported that homozygous *SMN2* deletion in ALS patients is not associated with survival or a decline in respiratory function.

More recently, Ishihara et al. [35] suggested that a decrease in the *SMN2* copy number may adversely affect the onset and prognosis of motor neuron diseases, including ALS and LMND, in the Japanese population. These authors reported that the *SMN2* copy number affects the survival time of patients with ALS (median survival: 0 copies, 34 months; 1 copy, 39 months; 2 copies, 44 months; 3 copies, 54 months; log-rank test, *p* < 0.05). Furthermore, *SMN2*-null cases (i.e., homozygous *SMN2* deletion or a loss of *SMN2*) were significantly more common in the LMND group (12.0%) than in the control group (4.8%) (odds ratio = 2.73, 95% confidence interval = 1.06–6.98, *p* < 0.05).

However, these discussions were all based on retrospective cohort studies. In addition, the discussions did not include any advanced molecular genetic studies after the discovery of *SMN1* and *SMN2*. Thus, prospective cohort studies incorporating the latest findings from molecular genetic research may provide more definitive conclusions about the relationship between homozygous *SMN2* deletion and motor neuron disease.

### 4.7. Limitations

In the present study, DBS samples with Ct values < mean + 1 SD were considered to carry at least one copy of *SMN2*, and further testing using PCR-RFLP or nucleotide sequence analysis was not performed. Therefore, the possibility of unrecognized false-negative results could not be completely excluded. However, such cases are expected to be very rare, and we consider them unlikely to contradict our statistical conclusions. Furthermore, time and labor constraints mean that confirmatory testing usually cannot be performed on all DBS samples; our limitations are therefore likely to be widely acceptable.

### 4.8. Conclusions

The present study of screening for homozygous *SMN2* deletion using real-time PCR revealed two important findings. First, the incidence of homozygous *SMN2* deletion in Japan is approximately 5%. Second, cross-reactive amplification with *SMN1* may result in false negatives in *SMN2* deletion screening. Importantly, this finding also suggests that cross-reactivity with *SMN2* may result in false-negative results in SMA-NBS, which screen for *SMN1* deletion.

## Figures and Tables

**Table 1:** PCR-RFLP summary of positive and ambiguous samples (*n* = 97).

Screening Assay by Real-Time PCR	Confirmatory Assay by PCR-RFLP		
	Homozygous *SMN2* Deletion	*SMN2* Retention	Total
Ct ≥ mean + 2SD (Group A)	45 (TP)	6 (FP)	51
Ct ≥ mean + 1SD and Ct < mean + 2SD (Group B)	4 (FN)	42 (TN)	46
Total	49	48	97

Ct: cycle threshold, FN: false negative, FP: false positive, PCR-RFLP: polymerase chain reaction–restriction fragment length polymorphism, SD: standard deviation, TN: true negative, TP: true positive.

**Table 2:** Nucleotide sequencing analysis summary of positive and ambiguous samples (*n* = 97).

Screening Assay by Real-Time PCR	Nucleotide Sequencing		
	Type I (with A)	Type II (with G)	Total
Ct ≥ mean + 2SD (Group A)	45 (TP)	6 (FP)	51
Ct ≥ mean + 1SD and <mean + 2SD (Group B)	4 (FN)	42 (TN)	46
Total	49	48	97

Ct: cycle threshold, FN: false negative, FP: false positive, PCR: polymerase chain reaction, SD: standard deviation, TN: true negative, TP: true positive.

**Table 3:** Screening assay summary of all samples (*n* = 1000).

Screening Assay	Homozygous *SMN2* Deletion	*SMN2* Retention	Total	
Screen-positive (Group A)	45 (TP)	6 (FP)	51	PPV 0.882
Screen-negative (Groups B and C)	4 (FN)	945 (TN) *	949	NPV 0.996
Total	49	951	1000	
	Sensitivity 0.918	Specificity 0.994		

Ct: cycle threshold, FN: false negative, FP: false positive, PCR: polymerase chain reaction, SD: standard deviation, TN: true negative, TP: true positive, PPV: positive predictive value, NPV: negative predictive value. * The 42 negative samples in Table 1 plus the 903 samples from Group C.

**Table 4:** Frequency of homozygous *SMN2* deletion.

	Homozygous *SMN2* Deletion	*SMN2* Retention	Total
Case number	49	951	1000
Percentage	4.9	95.1	100.0

## Data Availability

The data presented in this study are available upon request from the corresponding author.

## Supplementary Materials

The following supporting information can be downloaded at: https://www.mdpi.com/article/10.3390/genes16060712/s1, Table S1: Real-time PCR reaction mixture; Table S2: PCR-RFLP mixtures.

## Abbreviations

The following abbreviations are used in this manuscript:

ALS	Amyotrophic lateral sclerosis.
Ct	Cycle threshold.
DBS	Dried blood spot.
LMND	Lower motor neuron disease.
NBS	Newborn screening.
PCR	Polymerase chain reaction.
RFLP	Restriction fragment length polymorphism.
SD	Standard deviation.
SMA	Spinal muscular atrophy.
SMA-NBS	Newborn screening programs to detect SMN1 deletion.
SMN	Survival motor neuron.
