# Supplementary material for: Incidence of Homozygous SMN2 Deletion in Japan: Cross-Reactivity of SMN2 Primers with SMN1 Sequence Causes False Negatives in Real-Time PCR Screening
Source: Genes (Basel). 2025 Jun 16;16(6):712. doi: 10.3390/genes16060712 (PMC12193581; doi:10.3390/genes16060712)
Supplement: Supplementary file 1 [file genes-16-00712-s001.zip › genes-3672994-supplementary.pdf]

Table S1. Real-time PCR reaction mixture

Components	Volume (µL)
Distilled water	3
2× PCR Buffer for KOD FX Neo (Toyobo, Osaka, Japan)	12.5
KOD FX Neo (1.0U/µL) (Toyobo, Osaka, Japan)	0.5
2 mM dNTPs (Toyobo, Osaka, Japan)	5
cenSMNex7forw (10 pM)	0.25
cenSMNint7rev (10 pM)	0.25
20x EvaGreen® (Biotium, Hayward, CA, USA)	2.5
50x ROX Reference Dye (Thermo Fisher Scientific Inc., Waltham, MA, USA)	0.5
Dried Blood Spot (1.2 mm in diameter)	1 piece
Total	25

Table S2. PCR-RFLP mixtures

1. PCR mixture

Components	Volume (µL)
Distilled water	10.5
2× PCR Buffer for KOD FX Neo (Toyobo, Osaka, Japan)	25
KOD FX Neo (1.0U/µL) (Toyobo, Osaka, Japan)	1
2 mM dNTPs (Toyobo, Osaka, Japan)	10
R111 (8 pmol/mL)	1.5
X7-Dra (8 pmol/mL)	1.5
Dried Blood Spot (1.2 mm in diameter)	1 piece
Total	50

2. Enzyme digestion mixture

Components	Volume (µL)
Distilled water	6
10 × M Buffer (Takara Bio Inc., Shiga, Japan)	3
Dra I (15 U/µl) (Takara Bio Inc., Shiga, Japan)	3
PCR products	18
Total	30
